# An Improved Wildfire Smoke Detection Based on YOLOv8 and UAV Images

**DOI:** 10.3390/s23208374

**Published:** 2023-10-10

**Authors:** Saydirasulov Norkobil Saydirasulovich, Mukhriddin Mukhiddinov, Oybek Djuraev, Akmalbek Abdusalomov, Young-Im Cho

**Affiliations:** 1 Department of Computer Engineering, Gachon University, Seongnam 13120, Republic of Korea; saydirasulov@gachon.ac.kr; 2 Department of Communication and Digital Technologies, University of Management and Future Technologies, Tashkent 100208, Uzbekistan; mmuhriddinm@gmail.com (M.M.); odjuraev@gmail.com (O.D.); 3 Department of Artificial Intelligence, Tashkent State University of Economics, Tashkent 100066, Uzbekistan

**Keywords:** wildfire smoke detection, forest fire, UAV images, BiFormer, ghost shuffle convolution, remote sensing, deep learning, YOLOv8

## Abstract

Forest fires rank among the costliest and deadliest natural disasters globally. Identifying the smoke generated by forest fires is pivotal in facilitating the prompt suppression of developing fires. Nevertheless, succeeding techniques for detecting forest fire smoke encounter persistent issues, including a slow identification rate, suboptimal accuracy in detection, and challenges in distinguishing smoke originating from small sources. This study presents an enhanced YOLOv8 model customized to the context of unmanned aerial vehicle (UAV) images to address the challenges above and attain heightened precision in detection accuracy. Firstly, the research incorporates Wise-IoU (WIoU) v3 as a regression loss for bounding boxes, supplemented by a reasonable gradient allocation strategy that prioritizes samples of common quality. This strategic approach enhances the model’s capacity for precise localization. Secondly, the conventional convolutional process within the intermediate neck layer is substituted with the Ghost Shuffle Convolution mechanism. This strategic substitution reduces model parameters and expedites the convergence rate. Thirdly, recognizing the challenge of inadequately capturing salient features of forest fire smoke within intricate wooded settings, this study introduces the BiFormer attention mechanism. This mechanism strategically directs the model’s attention towards the feature intricacies of forest fire smoke, simultaneously suppressing the influence of irrelevant, non-target background information. The obtained experimental findings highlight the enhanced YOLOv8 model’s effectiveness in smoke detection, proving an average precision (AP) of 79.4%, signifying a notable 3.3% enhancement over the baseline. The model’s performance extends to average precision small (APS) and average precision large (APL), registering robust values of 71.3% and 92.6%, respectively.

## 1. Introduction

The escalation of the global warming trend has manifested notably in recent years, precipitating climate-induced drought and the emergence of El Niño events. Between 2013 and 2022, an annual mean of 61,410 wildfires transpired, comprising an average of 7.2 million acres. In the year 2022, a total of 68,988 wildfires raged, affecting 7.6 million acres of land. Remarkably, Alaska bore the brunt of this devastation, accounting for over 40% of the total acreage affected (equivalent to 3.1 million acres). As of 1 June 2023, the current year has witnessed approximately 18,300 wildfires, impacting a cumulative expanse of more than 511,000 acres. Notably, most of these wildfires are instigated by human activities, representing 89% of the average annual wildfire count from 2018 to 2022. Conversely, wildfires incited by lightning occurrences tend to exhibit comparatively larger scales and consume a more extensive acreage, accounting for approximately 53% of the mean property burned during the period spanning 2018 to 2022 [1].

Forest fires pose a serious hazard to both human lives and property, exerting a markedly harmful impact on the natural ecological balance of forest ecosystems. Furthermore, their occurrence remains unpredictable and engenders tough challenges regarding rescue operations [2,3]. As a result, the prevention of forest fires has consistently held a significant position in strategically establishing public infrastructure across diverse nations. In forest fire outbreaks, the representation of smoke typically precedes the actual ignition, with detectable pre-smoke indicators [4,5,6]. Timely and precise detection of wildfire-induced smoke holds immense significance, not solely for early forest fire alert systems and fighting measures but also for shortening the loss of human lives and property.

Traditional methods for monitoring forest fires involve manual observation through ground-based surveys and observation towers. Manual observation is sensitive to external factors such as logistical limitations, communication challenges, and weather, leading to inefficiencies. As a means of monitoring, observation towers possess limitations, including restricted coverage, areas with no surveillance coverage, and subsequent high maintenance expenses [7]. Despite its broad coverage, satellite-based monitoring [8] of forest fires faces limitations such as inadequate spatial resolution of satellite imagery, dependence on orbital cycles, susceptibility to weather and cloud cover interference, and low satellite numbers. Furthermore, achieving real-time forest fire monitoring using satellite systems is challenging.

Aerial monitoring has emerged as a productive method for forest fire surveillance [9], primarily using aircraft or unmanned aerial vehicles (UAV) and drones for surveillance. Nevertheless, this approach encounters substantial operational expenses due to the expansive expanse of forested landscape under consideration. Conventional methods of early forest fire detection predominantly rely on smoke and temperature-sensitive sensors, often in a combined configuration [10,11,12]. These sensors are engineered to detect airborne smoke particulates and swift escalations in ambient temperature, thereby facilitating fire detection. Notably, activating an alert is contingent upon achieving predetermined thresholds in either smoke concentration or ambient temperature. Despite their utility, these hardware-based sensors exhibit spatial and temporal constraints, compounded by challenges in maintenance after deployment. Consequently, it becomes evident that sensor-based solutions need to catch up in catering to the difficulties of real-time monitoring and preemptive detection and mitigation of forest fires within vast and complicated ecosystems, such as forests.

With the advancement of computer technology, there has been a shift towards more advanced approaches for detecting fire smoke, moving away from manual feature extraction methods. This newer paradigm predominantly revolves around surveillance systems positioned at observation points, capturing forest fire imagery or videos. Subsequently, manual extraction of features from these data sets is conducted, followed by the formulation of distinctive identifiers. This process is demonstrated in the work of Hidenori et al. [13], who used textural features of smoke to train a support vector machine model for identifying wildfire smoke. The efficacy of this approach is dependent on a sufficient number of training cases and the precision of feature extraction, both of which influence the recognition performance of the support vector machine. However, it is noteworthy that this technique engenders substantial data storage requirements and exhibits sluggish computational processing speeds. Fileonenko et al. [14] conducted smoke recognition by leveraging color and visual attributes inherent in smoke regions within surveillance videos. Exploiting the steadiness of the camera’s perspective, these researchers extracted smoke regions by computation of pixel edge roughness, subsequently employing background subtraction for identification. Nevertheless, this technique’s susceptibility to noise impairs its capability to achieve precision and rapid smoke detection. Tao and colleagues [15] worked on automating smoke detection using a Hidden Markov Model. They focused on capturing the changing characteristics of smoke areas in videos. They divided the color changes in consecutive frames into distinct blocks and used Markov models to classify each of these blocks. Despite these endeavors, this strategy still needs to be challenged by the intricacies of its operational setting. Traditional methods that use image or video analysis to detect forest fire smoke have achieved good results but also have some limitations. The underlying feature extraction process necessitates adept domain knowledge for feature selection, introducing the possibility of suboptimal design. Moreover, characteristics such as background, fog, cloud, and lighting can lead to reduced detection and recognition accuracy. Furthermore, these methods may not work as well in complex and changing forest circumstances.

With the rapid progress of deep learning techniques, researchers are increasingly using them for detecting forest fire smoke. Deep learning allows automatic detection and feature extraction through more complicated algorithms, leading to faster learning, better accuracy, and improved performance in dense forest conditions. For example, Zhang and colleagues [16] expanded their dataset by creating synthetic instances of forest fire smoke and used the Faster R-CNN framework for detection. This approach avoids the need for manual feature extraction but requires more computational resources. Another study by Qiang and team [17] used a dual-stream fusion method to detect wildfire smoke using a motion detection algorithm and deep learning. They achieved an accuracy of 90.6% by extracting temporal and spatial features from smoke images. However, there’s still a challenge in capturing feature information effectively from long sequences at the beginning. In the study by Filonenko et al. [18], various established convolutional classification networks, including VGG-19 [19], AlexNet [20], ResNet [21], VGG-16, and Xception, were utilized to classify wildfire smoke images. They employed Yuan’s dataset [22] of four smoke images for both training and validation. Their assessment of these model networks’ performance in recognizing smoke on this dataset highlighted Xception as the most effective detector. In another work, Li et al. [23] introduced an innovative technique called the Adaptive Depthwise Convolution module. This module dynamically adjusts the weights of convolutional layers to enhance the capture of features related to forest fire smoke. Their methodology yielded an accuracy of 87.26% at a frame rate of 43 FPS. Pan et al. [24] explored the deployment of ShuffleNet, coupled with Weakly Supervised Fine Segmentation and Faster R-CNN frameworks, for predicting the presence of fire smoke. However, due to the intricate nature of fire smoke and the high memory requirements for model training, the complexity of the task necessitated exceedingly robust hardware resources.

The extensive adaptability, rapidity, and precision of UAVs have led to their widespread integration in forest fire detection endeavors. UAVs can use their capacity to operate at low altitudes to capture high-resolution images of forested regions, enabling early fire identification. Moreover, UAVs demonstrate proficiency in navigating difficult and inaccessible terrains [25]. They can carry diverse cameras and sensors capable of detecting diverse spectral ranges, encompassing infrared radiation, which facilitates the discernment of latent heat sources beyond human visual perception. Furthermore, UAVs can be equipped with real-time communication systems, enabling quick responsiveness by firefighters and furnishing pertinent information about the fire’s parameters, positioning, and trajectory [26,27]. The collective attributes of UAVs render their deployment in forest fire detection increasingly pivotal, poised to assume an even more consequential role in the future of wildfire management.

Prior investigations into forest fire smoke detection have demonstrated the efficacy of various detection models, yielding favorable outcomes. Nevertheless, the complex background of forest environments and the difficulties linked to smoke feature extraction lead to numerous early detection challenges. Principally, forest imagery frequently encompasses both smoke and analogous background elements, such as clouds, water surfaces, and mist, which confound differentiation. The interplay of natural lighting fluctuations further compounds these issues, inducing image attribute alterations that impede downstream feature extraction and recognition processes. Moreover, precisely identifying nascent smoke instances remains formidable, given their dynamic characteristics and diminutive, indistinct shapes. Our framework employs an enhanced YOLOv8 model [28] for forest fire smoke detection. We initiated the model with pre-trained weights as foundational parameters for the underlying backbone network, subsequently adjusting network architecture parameters to optimize the conventional YOLOv8 model’s efficacy. Integrating this refined network architecture into a dataset relevant to forest fire smoke enabled precise recognition of perilous emissions such as smoke, including hazardous compounds.

The significant contributions of this study are as follows:

- We incorporate the Wise-IoU (WIoUv3) [29] method into the bounding box regression loss. This involves using a dynamic, non-monotonic approach to create a strategy for allocating gradient gains with improved rationality. WIoU v3 effectively adjusts gradient gains for samples of both high and low quality, resulting in enhanced precision in localization and an improved overall capacity for generalization in the model.
- We incorporate a dynamic sparse attention design named BiFormer [30] into the backbone network. This addition is known for its computational efficiency. By incorporating this mechanism, the model is better able to emphasize essential information within the feature map, ultimately improving its ability to detect objects.
- We employ GSConv [31] as a substitute for the conventional convolution within the neck layer, thereby establishing rapid pyramid pooling modules. This implementation expedites model convergence, facilitating the more expeditious amalgamation of smoke features with a reduced computational load when processing smoke images.
- In contrast to various prominent YOLO series models and an additional set of six conventional detection models, our approach showcases its evident superiority through comprehensive experimental outcomes.

The subsequent sections of this paper are structured as follows: Section 2 offers a presentation of the relevant literature. Section 3 outlines our dataset and the specific enhancements to YOLOv8. Section 4 provides a comprehensive account of the experimental findings and conducts a detailed performance analysis. Limitations and future work are discussed in Section 5. Ultimately, Section 6 serves to draw conclusions.

## 2. Related Works

Various approaches exist for smoke and fire detection, broadly categorized as follows: (a) vision-based methods and (b) sensor-based methods. This article specifically delves into vision-based strategies, crucial for outdoor settings where sensor deployment might be infeasible. Vision-based methods can be further divided into two distinct groups. The initial category entails feature extraction coupled with machine learning techniques, while the second category focuses on the utilization of deep neural networks.

### 2.1. Feature Extraction and Machine Learning-Based Approaches

In the context under consideration, the task of detecting smoke and fire entails the initial computation of a feature vector predicated on user-specified attributes. These attributes encompass color, motion, optical flow, and object morphology within the captured image. Subsequent to the computation of these features, they are subjected to analysis by a decision algorithm tasked with ascertaining the presence or absence of smoke or fire within the image. An approach for fire detection predicated on color and motion characteristics is expounded by Toreyin et al. [32]. In this particular study, alongside conventional color and motion attributes, the application of wavelet transform is incorporated for behavioral analysis and feature extraction within video content. This methodology necessitates the implementation of thresholding techniques to identify candidate fire areas. Furthermore, Chen et al. [33] introduce a method centered on the analysis of color and motion characteristics to detect smoke and fire. The technique in question involves the application of thresholds on RGB and HIS (hue, intensity, saturation) values, supplemented by a distinct threshold related to motion detection based on temporal variations in pixel color. Additionally, Dang-Ngoc et al. [34] employ image processing to discern fires within forested regions. In this work, an algorithm founded on the YCbCr color space, incorporating Y as luma (brightness), Cb as blue minus luma (B-Y), and Cr as red minus luma (R-Y) values, is introduced alongside conventional RGB values, aimed at heightening the accuracy of fire detection. Furthermore, Ghosh et al. [35] concurrently leverage color and motion attributes to detect smoke and fire. In this endeavor, fuzzy rules are employed to enhance classification performance. Conversely, Sankarasubramanian et al. [36] employ an edge detection algorithm to identify fire. Chen et al. [37] employs dynamic fire properties for fire area identification; however, instances involving objects resembling fire within the image might degrade the method’s performance. Lastly, Xie et al. [38] employ static and dynamic features in tandem for fire detection.

The important advantage inherent in these approaches lies in their minimal data requirements. Additionally, their incorporation of movement considerations serves to mitigate the misclassification of objects such as the sun as fire sources. Nonetheless, a drawback of these methods arises from their reliance on feature extraction methods anchored in attributes such as color. Consequently, these algorithms exhibit substantial error rates; for instance, an item such as a moving orange box might erroneously trigger a fire detection. Another noteworthy issue within this realm pertains to the necessity of fine-tuning pertinent thresholds, a labor-intensive process that often results in elevated false alarms. Moreover, the methods introduced in this domain grapple with the need for adept experience to appropriately design and configure suitable features.

### 2.2. Deep Learning-Based Approaches

In recent times, the adoption of deep learning techniques for the identification of smoke or fire in images has gained significant attention. Approaches grounded in artificial intelligence (AI) have effectively reduced the aforementioned shortcomings associated with feature-centric methodologies. For instance, Abdusalomov et al. [39] introduced a YOLOv3-based strategy for fire detection in indoor and outdoor environments, demonstrating its efficacy on a real-world image dataset and achieving an impressive accuracy of 92.8%. In another study, Khan et al. [40] proposed a hybrid approach that synergistically combined Convolutional Neural Networks (CNNs) and Recurrent Neural Networks (RNNs) for fire detection in smart urban settings, yielding high accuracy coupled with low false alarm rates. The domain of deep learning-based fire detection has also seen the utilization of Convolutional Neural Networks (CNNs), a class of deep neural networks adept at image processing tasks. Various researchers have proposed CNN-based fire detection systems, including seminal work such as the study conducted by Jeon et al. [41], presenting a CNN-centered fire detection methodology evaluated on indoor and outdoor fire image datasets, achieving an accuracy of 91%. Further contributing, Norkobil et al. [42] introduced a CNN-grounded fire detection system showcasing remarkable performance in video-based fire detection. Noteworthy studies in this field are explored in the following discourse.

In one study [43], a method focused on transfer learning is presented. It utilizes the pre-trained InceptionResNetV2 network to classify images as smoking or non-smoking. The effectiveness of this approach in predicting smoke and non-smoke images is assessed and compared with existing CNN methods using various performance metrics. Across a diverse and extensive new dataset, this method achieves accurate predictions of smoking or non-smoking images with a precision of 97.32%, accuracy of 96.87%, and recall of 96.46%. Talaat et al. [44] introduce an enhanced YOLOv8 model for fire detection using a dataset of fire and smoke images. The model incorporates a novel optimization function that reduces computational costs effectively. When compared to other studies, the adapted YOLOv8-based model demonstrates superior performance in minimizing false positives. Additionally, Liu et al. [45] propose a unique metric called “fire alarm authenticity”, which utilizes the duration of multiple smoke alarms’ alerts to determine fire location and severity. This criterion contributes to developing an algorithm for identifying alert sequences, validated through simulations involving real fires and false alarms.

The principal challenge associated with AI-driven methodologies resides in the demand for extensive training datasets and the time-intensive nature of the training process, compounded by limited oversight over the smoke and fire detection procedures. This concern is notably exacerbated by the lack of wide, standardized datasets exhibiting the requisite diversity. In the context of this study, a wide collection of datasets is curated to address these challenges and facilitate robust learning.

## 3. Materials and Methods

### 3.1. Overview of Wildfire Smoke Detection

**Figure 1:**
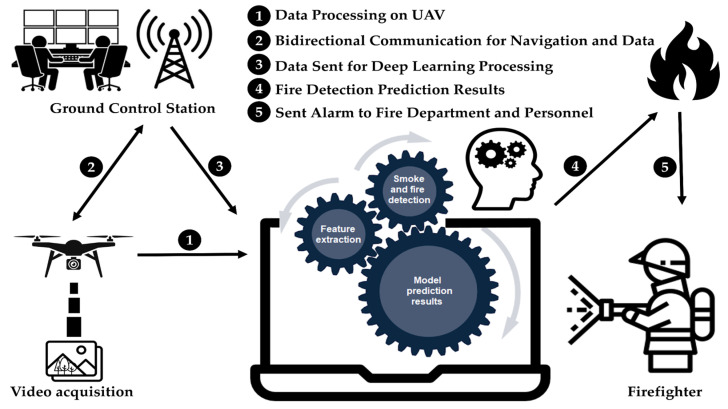
Overview of the proposed wildfire smoke detection system based on UAV images.

This section delineates the utilization of a deep learning model employed for the purpose of detecting wildfire smoke. Additionally, the dataset utilized for training purposes is explained. Prior to the commencement of the task, the requisite procedures, including navigation, model and algorithm selection, and system execution, must be successfully undertaken. As depicted in Figure 1, the camera onboard UAVs captures images or videos, which are then subjected to a sequence of operations encompassing preprocessing, feature extraction, smoke detection, and fire detection, ultimately culminating in the generation of predictive outcomes.

This research utilized UAV images and deep learning models to enhance the accuracy of early detection of forest fire smoke, even in varied weather conditions such as sunny, hazy, and cloudy atmospheres. We introduce an optimized YOLOv8 model along with a UAV image-based system for forest fire smoke detection. Usually, UAVs carry cameras that send images to a control station. At this station, an AI system is used to detect if there is smoke or fire. In this study, a method was developed that utilizes a deep neural network to accurately obtain precise localization of smoke regions, executed by a robust processor for rapid real-time image processing.

**Figure 2:**
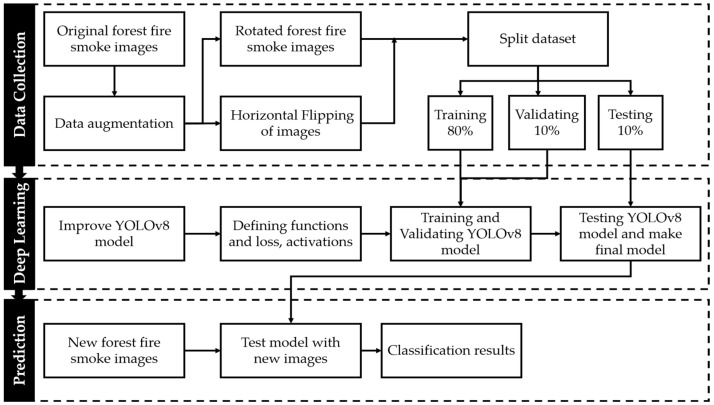
Overview of the proposed forest fire smoke detection system based on UAV images.

Upon obtaining the image and conducting essential preprocessing optimizations, the task necessitates the separation of pixels outlining the subject of interest from the surrounding image context. The extraction of features related to smoke and fire involved images captured under specific daytime and lighting circumstances. Aspects encompassing edges, corner points, motion, color attributes, luminance levels, and intensities were considered integral components of the feature extraction process. To conduct a comprehensive study of the segmented image and identify pivotal points of significance, the image underwent feature extraction procedures, thereby requiring the execution of relevant operations. The resultant processed image was subsequently inputted into a trained model to determine noticeable patterns that either affirm or reject the presence of smoke. The exact methodology of the proposed method is illustrated in Figure 2. In the subsequent phase, if the AI model produces a positive result, the system generates an alert using either the UAV platform or the control station. This alert prompts firefighting personnel to take the necessary actions.

### 3.2. Original YOLOv8

The YOLO model has achieved considerable acclaim within the domain of computer vision. Building upon this foundation, scholars have undertaken enhancements and incorporated novel modules into the methodology, giving rise to a multitude of classical models. Introduced by Ultralytics on 10 January 2023, YOLOv8 marks a significant advancement in this evolution. In contrast to earlier models such as YOLOv5 and YOLOv7, YOLOv8 is a cutting-edge and innovative model known for its improved detection accuracy and faster processing. The YOLOv8 network architecture comprises three main elements: the backbone, neck, and head [28].

The modified CSPDarknet53 [46] serves as the backbone network in YOLOv8, which results in five distinct scale features (denoted as B1–B5) through five consecutive downsampling stages. In the original backbone’s architecture, the Cross Stage Partial (CSP) module has been replaced with the C2f module. This new module, C2f, introduces a gradient shunt connection to enhance the flow of information within the feature extraction network while still maintaining a lightweight design. The CBS (Convolution, Batch Normalization, Silu) module is a composite element initially utilized in the YOLOv5 architecture for deep learning-based object detection tasks. This module combines three key components, namely: Convolution: Convolutional layers are employed to perform feature extraction from the input data. These layers apply convolutional operations to capture essential patterns and features within the data. Batch Normalization: Batch normalization is used to normalize the activations of the neural network at each layer. It helps stabilize and accelerate the training process by reducing internal covariate shifts. Silu Module: The Silu (Sigmoid Linear Unit) module, also known as the Swish activation function, is a type of activation function that introduces non-linearity into the network. It is known for its smooth gradient behavior, which aids in effective training. The CBS module, by incorporating these components, enhances the expressive power of the neural network and contributes to its ability to learn complex representations from the input data. This composite module is enabling more accurate and efficient object detection in a variety of applications. In the later stages of the backbone network, the spatial pyramid pooling fast (SPPF) module is utilized to adaptively generate output of a consistent size by pooling input feature maps. In comparison to the spatial pyramid pooling (SPP) structure [47], SPPF optimizes computational efficiency and reduces latency through a sequence of three consecutive maximum pooling layers.

Incorporating ideas from PANet [48], YOLOv8 introduces a PAN-FPN architecture into its neck component. Unlike the neck designs found in the YOLOv5 and YOLOv7 models, YOLOv8 brings about a modification by eliminating the convolution operation post up-sampling within the PAN structure. This alteration preserves the model’s initial performance while achieving a more streamlined configuration. Distinct feature scales within the PAN structure and FPN structure of the YOLOv8 model are denoted as P4–5 and N4-N5, respectively. Conventional FPN employs a top-down methodology to convey profound semantic details. However, while FPN enriches the merging of semantic information between B4–P4 and B3–P3, it may result in the loss of object localization information. To tackle this concern, PAN–FPN integrates PAN with FPN. By infusing PAN, the acquisition of location information is bolstered through the merging of P4–N4 and P5–N5, thereby facilitating an enhancement in the top-down pathway. This strategy orchestrates a comprehensive network structure that unifies both top-down and bottom-up components. Through feature fusion, it amalgamates surface-level positional insights and profound semantic details, thereby enriching the breadth and depth of features.

YOLOv8 employs a decoupled head architecture. This architecture features discrete branches for both object classification and the prediction of bounding box regression. Tailored loss functions are then applied to each task. Specifically, the task of bounding box regression prediction utilizes the CIoU [49] and distribution focal loss (DFL) [50]. Meanwhile, the classification task is supported by the binary cross-entropy loss (BCE loss). This deliberate design choice contributes to the enhancement of detection precision and accelerates the model’s convergence. YOLOv8 is distinct as an anchor-free detection model, simplifying the differentiation between positive and negative samples. Additionally, it incorporates the Task-Aligned Assigner [51] for dynamic sample allocation, thereby elevating both detection accuracy and the model’s robustness.

### 3.3. WIoUv3 Loss Function

Initially, the bounding box regression loss makes use of WIoUv3. Unlike the fixed focusing mechanism commonly employed by many traditional loss functions mentioned earlier, WIoU introduces a dynamic and non-monotonic focusing mechanism that goes beyond aspects such as overlap area, centroid distance, and aspect ratio. This mechanism aims to mitigate the influence of disproportionately large or extreme gradients that arise from outlier examples. WIoUv3 prioritizes the handling of samples of standard quality, thereby enhancing the model’s potential for abstraction and fortifying its general robustness. Tong et al. [29] introduced three variations of WIoU. While WIoUv1 was conceived with an attention-based predicted box loss, both WIoUv2 and WIoUv3 incorporated focusing coefficients to refine the approach.

WIoUv1 incorporates distance as an attention metric. Enhancing the model’s generalization capacity is facilitated by the reduction in the geometric measured penalty when the overlap between the object box and the predicted box falls within a designated range. The calculation formula for WIoUv1 is presented in Equations (1)–(3):

(1) $$L_{WIoUv1}=R_{WIoU}\times L_{IoU}$$

(2) $$R_{WIoU}=exp(\frac{{\left(b_{c_{x}}^{gt}-b_{c_{x}}\right)}^{2}+{\left(b_{c_{y}}^{gt}-b_{c_{y}}\right)}^{2}}{\left({c_{w}}^{2}+{c_{h}}^{2}\right)})$$

(3) $$L_{WIoU}=1-IoU$$

WIoUv2 is an extension of WIoUv1, incorporating the monotonic focus coefficient ${L*}_{IoU}$. This coefficient serves to effectively decrease the impact of straightforward samples on the loss value. However, to address the issue of slower convergence due to the decrease in ${L*}_{IoU}$ as $L_{IoU}$ decreases during model training, the average of $L_{IoU}$ is introduced to normalize ${L*}_{IoU}$. The mathematical formulation of WIoUv2 is provided in Equation (4):

(4) $$L_{WIoUv2}={\left(\frac{{L*}_{IoU}}{\bar{L_{IoU}}}\right)}^{\gamma }\times L_{WIoUv1},\gamma >0$$

The concept of outlier *β* is introduced by WIoUv3 to evaluate the quality of the anchor box, generating a non-monotonic focus factor r from this *β*, and then incorporating *r* into the established WIoUv1 formulation. A reduced *β* weight signifies superior anchor box quality, leading to a proportional reduction in the assigned *r* value, subsequently diminishing the impact of high-quality anchor instances in the overall loss function. Conversely, a larger β value signifies lower anchor box quality, leading to a reduced gradient gain allocation, which serves to mitigate adverse gradients stemming from low-quality anchor boxes. By dynamically allocating gradient gains, WIoUv3 optimizes the weighting of anchor boxes with varying qualities in the loss function, directing the model’s focus towards samples of average quality. This approach enhances the general implementation of the model through rational adjustments. Equations (5)–(7) present the formulations for WIoUv3. The parameters δ and α in Equation (6) are hyperparameters that can be tuned to align with specific model characteristics.

(5) $$L_{WIoUv3}=r\times L_{WIoUv1}$$

(6) $$r=(\frac{\beta }{{\delta \alpha }^{\beta -\delta }})$$

(7) $$\beta =\frac{{L*}_{IoU}}{\bar{L_{IoU}}}\in [0,+\infty ]$$

Through a comprehensive comparison of various mainstream loss functions, we ultimately introduce WIoUv3 as the chosen object bounding box regression loss. This decision is predicated on several factors. Firstly, WIoUv3 merges the merits of EIoU and SIoU, aligning with the design philosophy of exemplary loss functions. Utilizing a dynamic non-monotonic approach, WIoU v3 evaluates anchor box quality, with a specific focus on average-quality instances. This enhancement subsequently strengthens the model’s ability to precisely locate objects. In scenarios involving object detection through UAV images, the challenges posed by small objects are prominent. The adaptive adjustment of loss weights for small objects within WIoUv3 inherently contributes to the improved effectiveness of the model’s detection.

### 3.4. BiFormer Attention Mechanism

In images taken by UAVs, there are often complex backgrounds that can confuse detection models. These models might struggle to focus on what’s important and ignore the background. To address this, we’ve introduced an attention technique called BiFormer into the model’s core. It helps the model concentrate on the essential parts of the image and ignore the less important background. BiFormer does this by first figuring out which parts of the image matter the most, then focusing on those areas. This not only makes the model work better but also saves computer resources and makes the model more aware of what’s in the image. YOLOv8 is a type of CNN model. However, CNNs mainly focus on local features, which means they might miss out on understanding the broader relationships between different parts of an image. In contrast, transformers use an attention mechanism to estimate how much different pieces of data relate to each other, allowing them to capture global patterns effectively. This ability is especially valuable when dealing with complex and extensive datasets. The attention mechanism operates in this manner: First, the input data sequence [$a_{1},a_{2},a_{3},\cdots ,a_{T}$] is encoded to obtain [$x_{1},x_{2},x_{3},\cdots ,x_{T}$]. Then, three matrices—values V, keys K, and queries Q are produced using linear transformation matrices $W^{V}$, $W^{K}$, and $W^{Q}$. The calculation involves computing the dot product between every query and its connected key. Subsequently, the result is normalized and combined with matrix *V* through a weighted sum operation. To prevent the result’s gradient from vanishing, a term $\sqrt{d_{K}}$ is introduced, where $d_{K}$ represents the dimensionality of matrix K. The procedure for this attention process is outlined in Equation (8):

(8) $$Attention\left(Q,\;K,\;V\right)=softmax(\frac{{QK}^{T}}{\sqrt{d_{K}}})V$$

**Figure 3:**
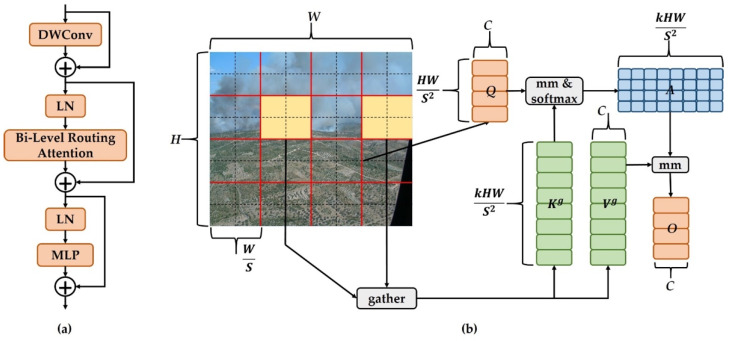
(**a**) Architecture of the BiFormer block; (**b**) Architecture of the Bi-Level Routing Attention block.

However, the typical attention mechanism comes with challenges such as high computational demands and substantial memory usage. When it comes to detection models used on UAV platforms, there are limitations in terms of available resources. Introducing a regular attention module directly into the model could take up a significant portion of these resources, leading to a decrease in the model’s speed for making predictions. To address these resource-related concerns, researchers have suggested a solution that involves using sparse queries focusing only on key-value pairs. Various related research has emerged from this approach, encompassing concepts such as expansive attention, deformable attention, and local attention. Nevertheless, these methods generally rely on manually designed content-independent sparsity and fixed patterns. To address these limitations, Lei Zhu and his team [31] introduced a creative solution named dynamic sparse attention, named the Bi-Level Routing Attention illustrated in Figure 3b.

As depicted in Figure 3b, the initial input feature map $X\in R^{H\times W\times C}$ is initially partitioned into S×S subregions, with each region containing $\frac{HW}{S^{2}}$ feature vectors. We modify the shape of X to yield $X^{r}\in R^{S^{2}\times \frac{HW}{S^{2}}\times C}$. Subsequently, the feature vectors undergo a linear transformation to yield three matrices, namely Q, K, and V. The mathematical formulas for these calculations are provided in Equations (9)–(11).

(9) $$Q=X^{r}W^{Q}$$

(10) $$K=X^{r}W^{K}$$

(11) $$V=X^{r}W^{V}$$

Next, the relationship of attention between different regions is established by constructing a directed graph and determining the connected regions for each given region. The specific process involves the following steps: For each region, the Q and V components are subjected to region averaging, producing the region-level counterparts $Q^{r}$ and $K^{r}\in R^{S^{2}\times C}$. Next, the dot product of $Q^{r}$ and $K^{r}$ is computed to generate the adjacency matrix $A^{r}\in R^{S^{2}\times S^{2}}$. This matrix gauges the correlation among different regions, and its formulation is presented in Equation (12).

(12) $$A^{r}={Q^{r}(K^{r})}^{T}$$

Thereafter, the matrix $A^{r}$ undergoes pruning, where the least relevant token in $A^{r}$ is removed, operating at a coarse level. This results in the retention of the top *k* most relevant regions within $A^{r}$, leading to the derivation of the routing index matrix denoted as $I^{r}\in N^{S^{2}\times k}$. The mathematical formulation for this process is depicted in Equation (13).

(13) $$I^{r}=topkIndex{(A}^{r})$$

Afterwards, a fine-grained token-to-token attention mechanism is employed. Within the context of a specific region i, this attention mechanism exclusively concentrates on the k routing regions, specifically indexed as $I_{\left(i,1\right)}^{r},I_{\left(i,2\right)}^{r},\ldots ,\;I_{\left(i,k\right)}^{r}\;$, thereby assembling all associated K and V tensors from these k regions to generate $K^{g}$ and $V^{g}$. The computational formulations for this process are presented in Equations (14) and (15).

(14) $$K^{g}=gather(K,I^{r})$$

(15) $$V^{g}=gather(V,I^{r})$$

In the concluding step, the aggregated $K^{g}$ and $V^{g}$ are subjected to an attention operation, and an additional term referred to as the local context enhancement *LCE(V)* is introduced to derive the resulting tensor O. The corresponding mathematical representation is provided in Equation (16).

(16) $$O=Attention(Q,K^{g},V^{g})+LCE(V)$$

The architecture of the BiFormer block is derived from the Bi-Level Routing Attention concept, illustrated in Figure 3a. Within this block, DWConv represents deep separable convolution, an operation that diminishes the model’s parameter count and computational load. LN signifies the application of layer normalization, a technique that expedites training and enhances the model’s ability to generalize. A multilayer perceptron is represented by the acronym MLP, and it serves to further fine-tune and modify attention weights in order to enhance the model’s emphasis on specific features. In Figure 3b, the addition symbol signifies the linkage of two feature vectors.

Incorporating the BiFormer block into the backbone network constitutes a key aspect of this research. This addition infuses the model with a dynamic attention mechanism that heightens its emphasis on vital object-related details, thereby augmenting the overall efficacy of object detection. To utilize the potential of this efficient attention mechanism, the BiFormer block is strategically positioned between B3 and B4, effectively supplanting the previously existing C2f block.

### 3.5. Ghost Shuffle Convolution (GSConv)

To enhance the efficiency of prediction computation towards the conclusion, the common practice within CNNs is to subject input images to a uniform transformation process in the backbone. This entails the progressive transfer of spatial information into the channels. However, at each stage of spatial compression and channel expansion, a certain degree of semantic information may be compromised. While channel-dense convolutional computation diligently retains inter-channel relationships, channel-sparse convolution severs these associations entirely. The GSConv method, in contrast, strives to preserve these connections to a significant extent while maintaining a lower time complexity.

Standard convolution (SConv) simultaneously applies distinct convolutional kernels across multiple channels, resulting in an augmented parameter count and a reduction in network speed as feature extraction intensifies. In contrast, depth-wise separable convolution (DWConv) consolidates the outcomes of discrete depth-wise convolutions via a 1 × 1 convolution kernel post-channel convolution. This strategy allows for substantial parameter reduction as feature complexity grows, thereby enhancing inference speed. Nevertheless, DWConv entails a trade-off by sacrificing a portion of semantic information during its operation, thereby compromising the model’s accuracy.

**Figure 4:**
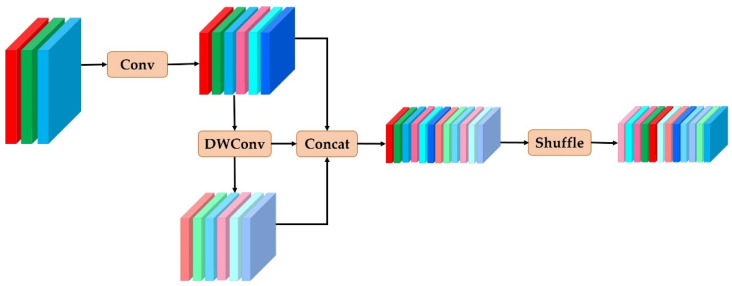
Architecture of the GSConv model.

The procedure of GSConv [31] is detailed in Figure 4, combining the merits of standard convolution and depth-separable convolution. It employs SConv and DWConv in tandem when processing input images of forest fire smoke. Unlike DWConv, GSConv refrains from severing the inter-channel connections entirely, opting instead to preserve these connections to a significant extent, thereby upholding model accuracy. The resulting features are merged and rearranged to amplify the non-linear representation. This is particularly valuable for smoke targets undergoing alterations due to fire and environmental conditions. The non-linear features offer an improved depiction of smoke’s deformation and expansion processes, thereby furnishing the model with enriched learning material and ultimately elevating its adaptability and resilience. The mathematical formulation is computed as outlined below in Equations (17) and (18):

(17) $$X_{c}=\sigma (bn(Conv2d(X_{input})))$$

(18) $$X_{out}=\delta (X_{c}⨁DWConv(X_{c}))$$

Here, Conv2d symbolizes the two-dimensional convolution applied to the input image $X_{input}$, bn denotes the normalization operation, σ signifies the activation function, ⨁ denotes the concatenation of the two convolution types, and the ultimate δ signifies shuffling, with the intent of deriving the last output $X_{out}$ through this shuffling process.

However, an all-encompassing integration of GSConv throughout all stages of the model would lead to a substantial escalation in the model’s layer computation, subsequently extending the inference duration required for rapid smoke target detection. As a result, it is advisable to restrict the use of GSConv to a single stage. Within the network architecture of YOLOv8, particularly in the backbone layer, where a significant amount of convolution is essential for extracting sufficient smoke-related features, preserving the substantial inter-channel correlation inherent to standard convolution is crucial. 

Through the replacement of standard convolution with GSConv, an endeavor focused on diminishing computational intricacies and parameter count, a more pronounced acceleration can be achieved in real-time execution. The incoming smoke image undergoes consecutive GSConv convolutions, and each shuffling operation adeptly amalgamates smoke feature maps from distinct channels with a diminished parameter count, thus approximating the outcome of standard convolution.

### 3.6. Forest Fire Smoke Dataset Collection

Thoroughly preparing an appropriate dataset stands out as a pivotal factor in the effective implementation of the algorithm, as elucidated in this paper. It’s important to highlight that the accuracy of deep learning models is inherently tied to the quality of images employed during the training and testing stages. Our analysis of forest fire smoke images has brought to light shortcomings in datasets used by vision-based systems, and existing open-access datasets have also demonstrated deficiencies. To empower our learning systems to discern various extents of forest fire smoke, we harnessed forest fire smoke images [10,52,53], along with wildland images [54] for non-wildfire scenarios, and augmented these with images sourced from the web. These datasets were acquired through the collection of pictures or videos taken by UAVs, aligning with the development of the forest fire smoke model optimized for UAV-based monitoring applications.

**Figure 5:**
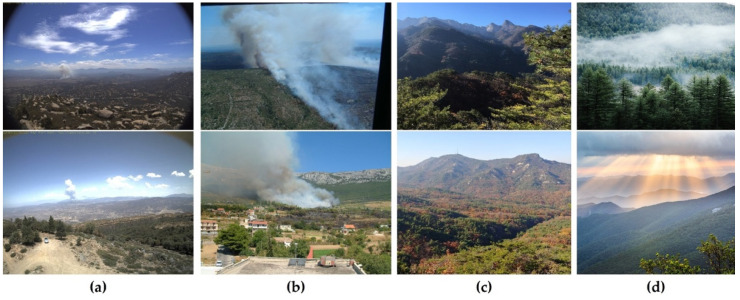
Illustrative samples from the forest fire smoke dataset include: (**a**) instances of small smoke with concentrated attention at the center and reduced attention at the edges; (**b**) varying sizes of large and medium smoke occurrences; (**c**) non-smoke pictures taken under diverse weather situations such as cloudy and sunny; and (**d**) instances with low smoke density, posing challenges in discerning attributes such as edges, textures, and color. This collection offers a representation of smoke scenarios encountered in natural environments.

The images gathered for the purpose of this research primarily comprise aerial photographs capturing instances of wildfire smoke alongside forest backgrounds. The dimensions of these images range from 2048 × 3072 to 512 × 512 pixels. These images portray recent global wildfire incidents. This diverse dataset enhances the algorithm’s capacity for generalization within intricate forest settings. Following a process of manual curation, we assembled a unified dataset encompassing 3200 images of forest fire smoke and 2800 images without wildfire smoke. The dimensions of all images were adjusted to 640 × 640 pixels. The specifics of these statements are provided in Table 1, and Figure 5 visually presents a selection of images from the wildfire smoke dataset. These images highlight the diversity in smoke appearance and dimensions within real-world environments, underscoring the challenges posed to conventional detection techniques.

Figure 5a displays images containing small-sized smoke instances, where the concentration is high at the center and low at the edges, presenting challenges in determining the smoke’s area. Conversely, Figure 5b shows medium and large wildfire smoke images. Figure 5c provides non-smoke images taken under diverse weather conditions, such as cloudy and sunny. Additionally, Figure 5d illustrates an image with a low smoke concentration where properties such as the edges of the smoke, texture, and color are not prominently discernible. Generally, the variation in smoke appearance and quantity in natural environments poses a challenge for conventional smoke detection systems. Consequently, the development of a wildfire smoke detection method capable of effectively identifying diverse smoke forms originating from natural sources is crucial. 

The effective performance of a deep learning model hinges on the availability of a substantial quantity of well-labeled training data. However, achieving reliable outcomes for wildfire smoke detection using such datasets can prove challenging due to issues such as overfitting, class imbalance, or insufficient data. Overfitting, characterized by a model’s failure to accurately capture visual patterns, is a potential concern. To address this, image data augmentation, involving the manipulation and reuse of existing images to enhance model accuracy, was employed as a remedy. Insights garnered from pertinent literature [55,56] underscore the significance of geometric modifications, encompassing flips and rotations, as valuable techniques for enhancing image data. By employing strategies such as rotation and horizontal flips [57,58], the forest fire smoke detection dataset was augmented experimentally, leading to an increase in the number of images. The performance of CNN models is notably responsive to the quantity and quality of image datasets utilized for training purposes.

Several modifications were introduced to each initial fire image to enhance the model’s capacity for generalization across the spectrum of preceding training images, enabling it to assimilate insights from a more extensive array of scenarios. These adaptations encompassed actions such as horizontal flipping and counterclockwise rotations of 60 and 120 degrees. Moreover, the training dataset was enriched by integrating images capturing non-smoke scenarios that share similarities with the environment, such as mountainous terrains, cloud formations, fog, and other comparable scenes. This initiative was undertaken to mitigate the occurrence of false positives.

To achieve our research goals, a dataset comprising 6000 images was utilized for the purpose of detecting forest fire smoke. This dataset was partitioned into a training subset containing 4800 images and a separate test subset comprising 1200 images. Only the training subset underwent data augmentation procedures, aiming to augment its volume. As outlined in Table 2, this approach led to a cumulative count of 30,000 images at the disposal for the task of identifying forest fire smoke.

## 4. Experimental Results

This section provides an elaborate description of the hyperparameter settings, the utilized test dataset, the experimental configuration, and the validation process employed to measure the effectiveness of the improved YOLOv8 model in identifying wildfire smoke in UAV photos. To ensure the reliability of the proposed methodology, all experiments were conducted under consistent hardware conditions. The experimentation was carried out on a self-assembled computer system with specific specifications, including Nvidia GeForce 1080 Ti graphics processing units, 32 GB of RAM, and a 9-core CPU running at 4.90 GHz [59], as specified in Table 3. The input images for the enhanced YOLOv8 model were drawn from a forest fire smoke dataset, each resized to dimensions of 640 × 640 pixels. The comprehensive evaluation encompasses a diverse range of facets, covering the experimental setup and design, YOLOv8 performance analysis, method impact assessment, model comparisons, ablation study, and visualization results. The table displaying the parameters utilized during the training of the model for detecting forest smoke has been included as Table 4 in the manuscript. This provides a clear overview of the training settings and configuration for this specific task.

### 4.1. Evaluation Metrics

In this study, a quantitative assessment of the proposed approach’s effectiveness was conducted using the well-established Microsoft COCO benchmarks (presented in Table 5), aligning with previous research endeavors [5,9,12,58,59,60]. A common metric for evaluating a classifier’s accuracy involves tallying the instances in which it correctly classifies an object. Conversely, a model’s recall denotes the ratio of its accurate predictions to the total count of ground truths, serving as a measure of its ability to correctly identify critical instances. A model with high recall can effectively identify a substantial portion of ground-truth items while maintaining precision by focusing on pertinent objects. An optimal model would indicate a false-negative rate of zero, a recall rate of one, and an accuracy rate of one. By comparing the results of the suggested method with ground-truth images pixel by pixel, followed by the calculation of precision and recall using Equations (19) and (20), the smoke detection method’s accuracy and recall rates were assessed.

(19) $${Precision}_{C_{ij}}=\frac{{TP}_{C_{ij}}}{{TP}_{C_{ij}}+{FP}_{C_{ij}}},$$

(20) $${Recall}_{C_{ij}}=\frac{{TP}_{C_{ij}}}{{TP}_{C_{ij}}+{FN}_{C_{ij}}},$$

The quantity of accurately identified smoke regions is denoted as ${TP}_{C_{ij}}$ (true positives), while instances of false positives stemming from the misclassification of non-smoke regions as smoke are indicated as ${FP}_{C_{ij}}$ (false positives). False negatives manifest when authentic smoke regions are erroneously classified as non-smoke regions, and they are denoted as ${FN}_{C_{ij}}$ (false negatives). The computation of the average precision (${AP}_{C_{ij}}$) was conducted using Equation (21) by considering these aforementioned values.

(21) $${AP}_{C_{ij}}=\frac{1}{m}\sum_{j=1}^{m}{Precision}_{C_{ij}},$$

The detection rate can be quantified as frames per second (*FPS*), representing the average rate of detection in terms of images processed per second. This calculation is based on the following formula:

(22) $$FPS=\frac{1}{t}$$

Here, *t* determines the average time for each image. This formula allows us to compute the frames per second metric, which is a crucial measure of the model’s real-time performance in processing images.

Additionally, we assessed the model’s complexity by quantifying the number of floating-point operations per second (FLOPS), which serves as a metric for gauging the computational workload of the model.

### 4.2. Quantitative Comparison

Comprehensive quantitative evaluations were conducted to calculate the effectiveness of our proposed method, utilizing documented Microsoft COCO benchmarks. These evaluations involved metrics such as precision, recall, and average precision (AP), calculated using Equations (19)–(21). To address the diverse range of smoke instances in our dataset, encompassing both small and large regions at varying distances, we systematically subjected object detectors, including various variants of the YOLO series, to thorough testing and comparison. This effort aimed to identify a robust method for accurately detecting smoke in wildfire circumstances.

Our study was centered on utilizing deep learning models for the purpose of detecting forest fire smoke, with the primary goal of minimizing the impact on forest ecosystems and safeguarding human lives. Following a thorough assessment of our dataset, we opted to employ YOLOv8 as our framework of choice, given its capability to swiftly identify smoke instances of varying sizes and orientations. It was observed that single-stage detectors, such as YOLOv8, were better suited for urgent scenarios and real-time deployment compared to the more intricate multi-stage object detectors prevalent in the field. The proposed model for forest smoke detection, built upon the foundation of YOLOv8, reaches notable enhancements across several performance metrics, including AP, AP50, AP75, APS, APM, and APL, when contrasted with alternative object detection approaches.

In order to comprehensively evaluate the strengths of the proposed methodology, a comparative analysis was conducted against a range of multi-stage object detection techniques, which encompassed MegDet [61], Faster R-CNN [16], Fast R-CNN [62], Mask R-CNN [63], Libra-R-CNN [64], DeNet [65], Cascade R-CNN [66], and CoupleNet [67]. Additionally, the assessment incorporated various single-stage object detection methods, including YOLOv3 [46], YOLOv4 [68], YOLOv5 [69], YOLOv7 [70], YOLOv8 [28], FSAF [71], M2Det [72], EfficientDet [73], RefineDet [74], SSD [75], NAS-FPN [76], DeepSmoke [77], RFBNet [78], and RetinaNet [79]. Elaborated insights into the performance of the enhanced YOLOv8 model and the multi-stage object detectors on the forest fire smoke dataset are presented in Table 6. Consistency was maintained throughout the comparisons by utilizing the identical set of training and testing images from the custom wildfire smoke dataset. Furthermore, Table 7 offers a comparative evaluation of the improved YOLOv8 model against other single-stage object detectors using the same dataset. In terms of forest fire smoke detection, our proposed model stands out favorably in comparison to other object detection methodologies. In Table 6, it is evident that Mask R-CNN [63] and Cascade-R-CNN [66] achieve the second and third best results, boasting AP50 scores of 77.6% and 80.4%, respectively. Conversely, Libra-R-CNN [64] and Denet [65] exhibit lower performance, yielding scores of 65.5% and 66.3%, respectively. Our proposed method achieves noteworthy results, demonstrating an average precision of 78.5% for small objects and an impressive 92.6% AP for large objects. Typically, single-stage object detectors tend to exhibit higher precision results compared to multiple-stage object detectors. As depicted in Table 7, versions of the YOLO object detector [28,70] achieve the second and third best AP results, registering scores of 76.1% and 75.2%, respectively. In contrast, single-stage detectors such as M2Det [72] and FSAF [71] demonstrate comparatively lower AP performance, with 60.2% and 60.5% in the results, respectively.

### 4.3. Qualitative Evaluation

**Figure 6:**
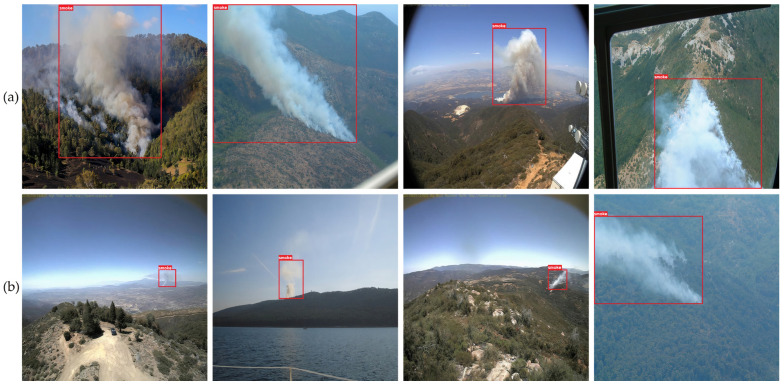
Example of qualitative evaluation of the forest fire smoke detection model: (**a**) large-size smoke; (**b**) small-size smoke.

Apart from the quantitative assessment conducted to evaluate the proposed methodology’s efficacy in detecting smoke arising from forest fires, a qualitative investigation was also undertaken. For this purpose, a selection of eight images was made from the dataset. Among these, four images depicted substantial smoke plumes arising from forest fires, while the remaining four showed smaller, spontaneous smoke plumes. Employing the optimized YOLOv8 model yielded consistent and dependable outcomes across both categories, as illustrated in Figure 6. The presented images portrayed a diverse range of scenarios and conditions, encompassing instances of smoke dispersing in various directions.

Numerous methodologies outlined in the existing literature have encountered challenges in effectively detecting smoke from minor wildfire incidents in images. To address this, we curated a collection of photographs capturing forest fire smoke on varying scales, aiming to augment the dataset and enhance the precision of smoke detection. In Figure 6b, smoke images characterized by smaller dimensions are showcased. In order to identify diminutive moving entities while retaining intricate attributes, we adopted a strategy influenced by previous work [9]. This approach involves amalgamating a feature map originating from a preceding layer with a high-scale feature map. The extensive feature map holds the capacity to discern smoke pixels exhibiting diverse dimensions, as it combines positional information from lower strata with intricate characteristics derived from upper layers.

Figure 6 visually illustrates the efficacy of the proposed methodology for forest fire smoke identification, employing the enhanced YOLOv8 model, in a diverse array of forest backgrounds. The robustness of the proposed technique underwent verification through assessments involving both substantial and minute smoke images. Timely detection of smoke is pivotal for forest fire prevention and containment efforts. Even a minor hint of smoke can activate a catastrophic forest fire if left unchecked, endangering human lives, natural resources, and ecosystems. Moreover, the proposed approach demonstrates remarkable precision in detecting minute smoke patches within images.

The outcomes of our study demonstrate the effective capacity of the proposed method to significantly reduce instances of false detections. This efficacy translates to expedited suppression and prompt response durations across a spectrum of forest fire smoke scenarios, irrespective of their orientation, morphology, or scale. Traditional visual smoke and fire detection systems tend to misclassify slight amounts of smoke sharing analogous color and intensity attributes with the surrounding environment as actual smoke.

### 4.4. Ablation Study

In order to conduct ablation analyses aimed at evaluating the efficacy of different bounding box regression loss modules, we substituted the WIoU loss module with the Generalized-IoU (GIoU), Distance-IoU (DIoU), and Complete-IoU (CIoU) loss modules. The GIoU loss was introduced as a remedy for the deficiencies observed in the original IoU loss. In comparison to the IoU loss, the GIoU loss exhibits enhanced dynamic behavior, enabling it to capture the spatial arrangement between two bounding boxes even when the IoU is equal to zero. However, the GIoU loss is not without its limitations. For example, in scenarios where a containment relationship exists between two bounding boxes, the GIoU loss regresses to the IoU loss, failing to discern the relative positioning of the boxes. Furthermore, in cases where a significant vertical directional disparity occurs between the two boxes, the GIoU loss demonstrates instability, potentially impeding convergence during the optimization process. The DIoU loss, introduced as an extension of the IoU loss, incorporates a supplementary penalty term related to the distance between the centers of two bounding boxes. This inclusion facilitates faster model convergence during optimization. While the DIoU loss does alleviate the gradual convergence issue associated with the GIoU loss to some degree, it retains limitations in accurately characterizing the overlap information between the two bounding boxes. Furthermore, even with the DIoU loss, when the center points of the two boxes coincide entirely, both the GIoU and DIoU losses revert to the IoU loss. The CIoU loss, an enhanced version of the DIoU loss, integrates the aspect ratio characteristics of two bounding boxes. This augmentation enables a more accurate representation of the spatial distance and alignment between the boxes, consequently advancing the effectiveness and efficiency of regression. Nevertheless, it’s worth noting that the aspect ratios employed in the CIoU loss are relative measurements, introducing a certain level of inherent uncertainty.

In order to ascertain the effectiveness of the improved algorithm, the present research integrated the WIoUv3 as the bounding box regression loss within the YOLOv8 model and conducted a comprehensive analysis using the custom smoke dataset. The outcomes, quantified through metrics such as AP, AP50, AP75, APS, APM, and APL, are presented in Table 8 for evaluation purposes.

Table 8 presents the outcome of ablation experiments, showcasing a comparison between the enhanced YOLOv8 model and the incorporation of GIoU and DIoU losses into the YOLOv8 model. When compared with the original YOLOv8 algorithm, the inclusion of GIoU and DIoU losses led to diminished accuracy, reduced recall, and lower AP scores, all while intensifying the computational load on the model. The experimental findings conclusively highlight that the model achieves its optimum detection performance by employing WIoUv3 as the bounding box for regression loss. Conversely, the utilization of WIoUv3 for bounding box regression yielded improved average precision scores.

Additionally, this research encompasses ablation experiments designed to assess the impact of the GSConv and BiFormer modules on enhancing the accuracy of the proposed YOLOv8 smoke detection model. Four distinct ablation experiments were conducted, comprising YOLOv8, YOLOv8 with GSConv, YOLOv8 with BiFormer, and YOLOv8 with both GSConv and BiFormer. The outcomes of these ablation experiments are presented in Table 9, revealing that the introduced modifications have the potential to elevate the performance of the YOLOv8 model.

Ablation studies have demonstrated that despite the robustness of the YOLOv8 object detection model, its performance can be suboptimal in certain scenarios. These findings suggest that the integration of GSConv and BiFormer into the network architecture of YOLOv8 could lead to substantial improvements in model accuracy.

## 5. Limitations and Future Works

In contrast to various other applications of computer vision, such as facial recognition, defect identification, and lane tracking, the task of forest fire smoke detection presents unique challenges. This task is compounded by the dynamic and irregular nature of smoke plumes, as well as the presence of numerous environmental variables within the complex forested landscape, including factors such as haze and cloud cover. Timely and accurate detection of even minor fires is of paramount importance, as they can rapidly escalate into large-scale disasters with devastating consequences if not promptly identified. Leveraging computer vision technology to replace human surveillance offers a highly effective means of addressing these challenges, primarily due to its inherent advantages.

However, it’s important to acknowledge that while the proposed forest fire smoke detection method has demonstrated success, it does have specific limitations. Notably, its sensitivity to atmospheric conditions such as fog, haze, and clouds poses a significant challenge, as these conditions can sometimes mimic the appearance of smoke. Additionally, situations where pixel values resemble those of a smoke plume in cloudy or hazy environments present a substantial obstacle. To address these limitations and enhance the accuracy of smoke detection, we intend to invest in technology capable of distinguishing between various cloud sizes and types of smoke. These enhancements aim to improve the model’s predictive performance by expanding the training dataset and extracting more informative features. One potential avenue for further exploration involves the incorporation of modules for determining the size and shape of smoke plumes. It’s worth noting that our analysis was primarily conducted during daylight hours. Consequently, a focus of future research will be on evaluating the model’s effectiveness in detecting wildfires during nighttime conditions. Based on our findings, it’s important to recognize that smoke detectors may not perform as effectively as fire alarms in low-light environments.

Our forthcoming endeavors will be dedicated to mitigating the issue of excessive false positives generated by the model, particularly in challenging scenarios such as those characterized by low-altitude cloud cover and haze. Given the temporal and environmental patterns associated with fire occurrences, particularly in specific geographical areas and during particular months, we aim to enhance predictive accuracy by incorporating supplementary contextual information such as fire location, date, and historical meteorological data. Furthermore, we recognize the need to adapt the proposed method for compatibility with edge devices. To address this challenge, we intend to optimize the model’s size without compromising its performance. Leveraging distillation techniques for the training of a more compact deep network, such as YOLOv8n, offers a promising avenue for constructing a model tailored for edge computing while upholding the same level of performance exhibited by our current model.

## 6. Conclusions

The challenge of achieving robust performance in wildfire smoke detection algorithms arises from the lack of suitable training images, leading to complications such as overfitting and data imbalance. In this study, we present an improved YOLOv8 model customized for wildfire smoke detection under complicated forest conditions. As shown in Table 9, these improvements, which include features such as GSConv and BiFormer, lead to remarkable results with an AP of 79.4%, an AP50 of 87.1%, and an AP75 of 82.4%. Consequently, the improvements contribute to an improved AP, AP50, and AP75, representing increases of 3.3%, 2.8%, and 5%, respectively. In the ablation analysis focused on bounding box regression, the consistently superior performance of WIuOv3 is evident with an AP50 of 85.1%, outperforming GIuO and DIoU with AP50 values of 84.6% and 84.5%, respectively. The experimental results highlight that the optimized YOLOv8 model outperforms both the state-of-the-art models and the multilevel models for object detection on the specific smoke image dataset, achieving an APS of 71.3% and an APL of 92.6%, respectively. While YOLOv8 achieves the second-best performance on AP75 and APL with 77.4% and 89.3%, respectively, conventional wildland fire smoke detection sensors are reaching their limits in terms of coverage of a limited area and ability to detect fires simultaneously. The refined YOLOv8 approach alleviates these limitations and enables wildfire smoke detection with geographic and material attributes. 

Enhancing the diversity of wildfire smoke pictures is critical for advances in wildfire smoke detection in natural environments. Thus, our prospective study will concentrate on collecting a variety of images of smoke from wildfires and using techniques to improve these images. We will also look for ways to speed up the detection process without losing accuracy by making the model smaller. In addition, the development of robust algorithms for use in real time under different environmental conditions is needed. In addition, the integration of multimodal data sources, such as satellite imagery and weather data, can improve the accuracy and reliability of recognition systems. Emphasizing these aspects would not only improve early detection of wildfires but also contribute to effective disaster mitigation and management strategies, thereby protecting ecosystems and human lives.

## Figures and Tables

**Table 1:** Forest fire smoke dataset and its specification.

Dataset	Smoke Images				Non-Smoke Images				Total
	Google	Kaggle	Flickr	Bing	Google	Kaggle	Flickr	Bing	
Forest Fire Smoke	300	2500	100	300	150	2400	100	150	6000

**Table 2:** Data augmentation on the wildfire smoke dataset.

Forest Fire Smoke	Training Images			Testing Images	Total
	Original Images	Rotated Images	Flipped Images	Original Images	
Smoke images	2600	5200	7800	650	16,250
Non-smoke images	2200	4400	6600	550	13,750
Total	4800	9600	14,400	1200	30,000

**Table 3:** Specifications of hardware and software.

Items	GPU	CPU	RAM	Motherboard	OS	Storage
Specifications	GPU 2-GeForce 1080	Intel Core 9 Gen i7-9700 k (4.90 GHz)	DDR4 32 GB (DDR4 16 GB × 2)	ASUS PRIME Z390-A STCOM	Ubuntu Desktop (version: 18.0.4 LTS)	SSD: 512 GB/HDD: TB (2 TB × 2)

**Table 4:** Hyperparameters for training forest fire smoke detection method.

Training Hyperparameters	Details
Epoch	200
Image size	640 × 640
Batch size	32
Learning rate	0.001

**Table 5:** Microsoft’s COCO benchmarks for object detection methods.

AP	AP_50_	AP at IoU = 0.5
AP	AP_75_	AP at IoU = 0.75
AP at different levels	AP_S_	AP_0.5_ for small area: area < $32^{2}$
	AP_M_	AP_0.5_ for medium area: $32^{2}<$ area < $96^{2}$
	AP_L_	AP_0.5_ for large area: area > $96^{2}$

**Table 6:** Comparison results between the proposed method and multiple-stage object detectors.

Models	AP	AP_50_	AP_75_	AP_S_	AP_M_	AP_L_	FPS
MegDet [61]	64.2	73.1	67.2	54.8	63.5	78.1	-
Faster R-CNN [16]	65.7	72.6	67.3	55.7	64.4	76.3	-
Fast R-CNN [62]	63.5	70.3	64.4	53.1	62.3	75.2	-
Mask R-CNN [63]	69.3	77.6	73.0	60.5	68.2	81.0	-
Libra-R-CNN [64]	54.4	65.5	61.2	45.2	53.6	70.4	-
DeNet [65]	57.1	66.3	60.5	47.3	58.4	72.4	-
Cascade R-CNN [66]	72.0	80.4	76.2	63.9	71.1	85.6	-
CoupleNet [67]	60.6	67.3	62.6	50.4	60.0	72.5	-
The proposed	79.4	87.1	82.4	71.3	78.5	92.6	167

**Table 7:** Comparison results between the proposed method and single-stage object detectors.

Models	AP	AP_50_	AP_75_	AP_S_	AP_M_	AP_L_	FPS
YOLOv3 [46]	69.4	77.2	70.3	61.0	68.7	80.5	33
YOLOv4 [68]	71.5	79.4	73.5	62.3	70.1	83.7	37
YOLOv5 [69]	72.3	80.0	74.2	64.6	71.4	85.4	160
YOLOv7 [70]	75.2	83.2	76.1	68.0	74.5	88.2	163
YOLOv8 [28]	76.1	84.3	77.4	69.5	75.6	89.3	168
FSAF [71]	60.5	70.7	64.7	52.6	60.1	76.1	24
M2Det [72]	60.2	70.4	64.5	52.3	59.4	75.6	28
EfficientDet [73]	72.6	79.2	75.4	64.5	71.3	84.7	30
RefineDet [74]	70.0	77.3	72.7	61.7	68.5	83.3	63
SSD [75]	65.3	73.5	67.1	56.6	65.6	78.0	84
NAS-FPN [76]	63.2	73.0	67.3	55.1	62.7	77.1	22
DeepSmoke [77]	73.4	80.6	75.2	65.4	72.4	87.0	36
RFBNet [78]	64.2	70.1	65.0	53.2	61.0	74.8	27
RetinaNet [79]	67.0	74.7	69.1	58.5	65.1	70.5	69
The proposed	79.4	87.1	82.4	71.3	78.5	92.6	167

**Table 8:** Comparison results of the ablation study for bounding box regression.

Model	Bounding Box Regression				Evaluation Metrics							
	WIoUv3	GIoU	DIoU	AP	AP_50_	AP_75_	AP_S_	AP_M_	AP_L_	FPS	GFLOPS	Latency
YOLOv8	×	×	×	76.1	84.3	77.4	69.5	75.6	89.3	168	107.3	13 ms
	√	×	×	76.9	85.1	78.3	70.3	76.4	90.1	166	106.5	9 ms
	×	√	×	76.4	84.6	77.8	69.8	75.9	89.7	167	106.3	10 ms
	×	×	√	76.3	84.5	77.7	69.7	75.9	89.6	168	106.7	11 ms

**Table 9:** Comparison results of the ablation study for various modules.

Model	Modules		Evaluation Metrics								
	GSConv	BiFormer	AP	AP_50_	AP_75_	AP_S_	AP_M_	AP_L_	FPS	GFLOPS	Latency
YOLOv8 + WIoUv3	×	×	76.9	85.1	78.3	70.3	76.4	90.1	166	106.5	9 ms
	√	×	78.3	86.3	80.5	70.9	77.6	91.5	168	106.7	9 ms
	×	√	78.0	85.9	80.2	70.7	77.3	91.2	165	105.8	9 ms
	√	√	79.4	87.1	82.4	71.3	78.5	92.6	167	103.5	8 ms

## Data Availability

Not applicable.
